# Hair-sparing whole brain radiotherapy with volumetric arc therapy in patients treated for brain metastases: dosimetric and clinical results of a phase II trial

**DOI:** 10.1186/1748-717X-9-170

**Published:** 2014-07-29

**Authors:** Annemieke De Puysseleyr, Joris Van De Velde, Bruno Speleers, Tom Vercauteren, Anneleen Goedgebeur, Tom Van Hoof, Tom Boterberg, Wilfried De Neve, Carlos De Wagter, Piet Ost

**Affiliations:** 1 Department of Radiotherapy and Experimental Cancer Research, Ghent University, De Pintelaan 185, Ghent, Belgium; 2 Department of Anatomy, Ghent University, De Pintelaan 185, Ghent, Belgium; 3 Department of Radiotherapy, Ghent University Hospital, De Pintelaan 185, Ghent, Belgium

## Abstract

**Purpose:**

To report the dosimetric results and impact of volumetric arc therapy (VMAT) on temporary alopecia and hair-loss related quality of life (QOL) in whole brain radiotherapy (WBRT).

**Methods:**

The potential of VMAT-WBRT to reduce the dose to the hair follicles was assessed. A human cadaver was treated with both VMAT-WBRT and conventional opposed field (OF) WBRT, while the subcutaneously absorbed dose was measured by radiochromic films and calculated by the planning system. The impact of these dose reductions on temporary alopecia was examined in a prospective phase II trial, with the mean score of hair loss at 1 month after VMAT-WBRT (EORTC-QOL BN20) as a primary endpoint and delivering a dose of 20 Gy in 5 fractions. An interim analysis was planned after including 10 patients to rule out futility, defined as a mean score of hair loss exceeding 56.7. A secondary endpoint was the global alopecia areata severity score measured with the “Severity of Alopecia Tool” (SALT) with a scale of 0 (no hair loss) to 100 (complete alopecia).

**Results:**

For VMAT-WBRT, the cadaver measurements demonstrated a dose reduction to the hair follicle volume of 20.5% on average and of 41.8% on the frontal-vertex-occipital medial axis as compared to OF-WBRT. In the phase II trial, a total of 10 patients were included before the trial was halted due to futility. The EORTC BN20 hair loss score following WBRT was 95 (SD 12.6). The average median dose to the hair follicle volume was 12.6 Gy (SD 0.9), corresponding to a 37% dose reduction compared to the prescribed dose. This resulted in a mean SALT-score of 75.

**Conclusions:**

Compared to OF-WBRT, VMAT-WBRT substantially reduces hair follicle dose. These dose reductions could not be related to an improved QOL or SALT score.

## Introduction

Whole brain radiotherapy (WBRT) increases survival in patients with brain metastases [1-3], but has also been shown to reduce Quality of Life (QOL) by increasing drowsiness, leg weakness and hair loss [4]. Hair loss is typically observed in all patients undergoing WBRT [5] and was reported as one of the main factors reducing QOL scores when WBRT is used in the prophylactic setting in small cell lung cancer [6].

The standard WBRT technique applies 2 lateral opposed fields (OF-WBRT) with a margin around the brain including the hair follicles, which are located approximately 5 mm below the scalp [7]. Irradiation-induced epilation is due to high susceptibility of anagen follicles to radiation [8]. Complete hair regrowth generally occurs 2–4 months after irradiation [8], which often exceeds the life expectancy in poor prognostic patients. Therefore, significant gains in QOL might be obtained by developing a hair sparing WBRT technique. Recent technological improvements in patient positioning and radiotherapy treatment planning may allow us to treat the whole brain with reduced margins, allowing better sparing of the scalp [9-11]. Furthermore, the use of arc techniques may minimize the follicle dose, as the reduction of surface dose is then distributed over the length of the arc [12].

The purpose of this study was to investigate the impact of VMAT-WBRT on temporary alopecia and hair loss related QOL. The dose-effect relation of temporary alopecia is, however, poorly investigated and, to our knowledge, the available data remain limited to permanent alopecia [12,13]. Furthermore, plan optimization and evaluation for the hair follicle volume is impeded by the known inaccuracies of the treatment planning system in calculating build-up doses [14]. In order to overcome these issues, our study combines a dosimetric study and a prospective phase II trial for the treatment of patients with brain metastases, relating the measured and computed dose reductions to temporary alopecia and hair loss related QOL.

## Methods

### Dosimetric evaluation

In order to quantify the potential of VMAT-WBRT to reduce the dose to the hair follicles and to evaluate the treatment planning system’s accuracy in predicting these values, a human Thiel-embalmed cadaver [15] implanted with 13 radiochromic films in the scalp was treated with both OF- and VMAT-WBRT (Ghent University Hospital Institutional Review Board reference EC UZG 2012/401).

#### *Treatment techniques*

**Figure 1:**
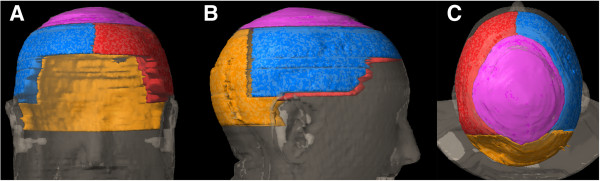
**The hair follicle volume was defined as the tissue underlying the skin up to the outer table of the skull.** An automated script was written in Pinnacle, version 9.0 (Philips Medical Systems, Andover, MA, US), to automatically contour this volume with the creation of 4 subvolumes representing the different areas of the scalp: top (vertex, pink), back (posterior aspect of the scalp, orange), left (left profile of the scalp, blue) and right (right profile of the scalp, red). **Panel A**: frontal view, **panel B**, lateral view, **panel C**: cranial view.

The OF-WBRT plan consisted of two lateral opposed beams, with the gantry at 90 and 270 degrees with the field borders set at 2 cm beyond the bony skeleton in the superior, anterior and posterior direction. The collimator was rotated to allow the inferior border to parallel the base of skull. The inferior border was set from the bony canthus to the C1-2 interverebral space, covering the base of skull with a 1-cm margin. The VMAT-WBRT plan used a single arc of 352° (from -176° to +176°) without gaps in delivery. Both techniques employed 6 MV photons from a linear accelerator equipped with an MLCi2 multileaf collimator (Elekta Synergy, Elekta, Crawley, UK). The clinical target volume was the whole brain (to the inner table of the skull and 1 cm inferior for the foramen magnum), delineated on a contrast edical Systems, Tokyo, Japan) and expanded with 2 mm in all directions creating a planning target volume (PTV) for both techniques. The hair follicle volume was defined as the tissue underlying the skin up to the outer table of the skull. An automated script was written in Pinnacle, version 9.0 (Philips Medical Systems, Andover, MA, US), to automatically contour this volume with the creation of 4 subvolumes representing the different areas of the scalp (Figure 1): top (vertex), back (posterior aspect of the scalp), left (left profile of the scalp) and right (right profile of the scalp) [16]. A median dose of 20 Gy (5 fractions) was prescribed to the PTV according the ICRU recommendations (95% - 107%) [17] while the dose to the hair follicle volume was reduced maximally without compromising the dose to the brain. Optimization and dose calculation were first performed using the VMAT-extended GRaTiS program [18,19] with final dose calculations in Pinnacle.

During treatment, the cadaver was positioned on a personalized head support and immobilized with a thermoplastic mask (Marcomedics, Waddinxveen, The Netherlands). The positioning of the brain was corrected using cone beam CT, with co-registration based on the bony anatomy [20].

#### *Radiochromic film dosimetry*

**Figure 2:**
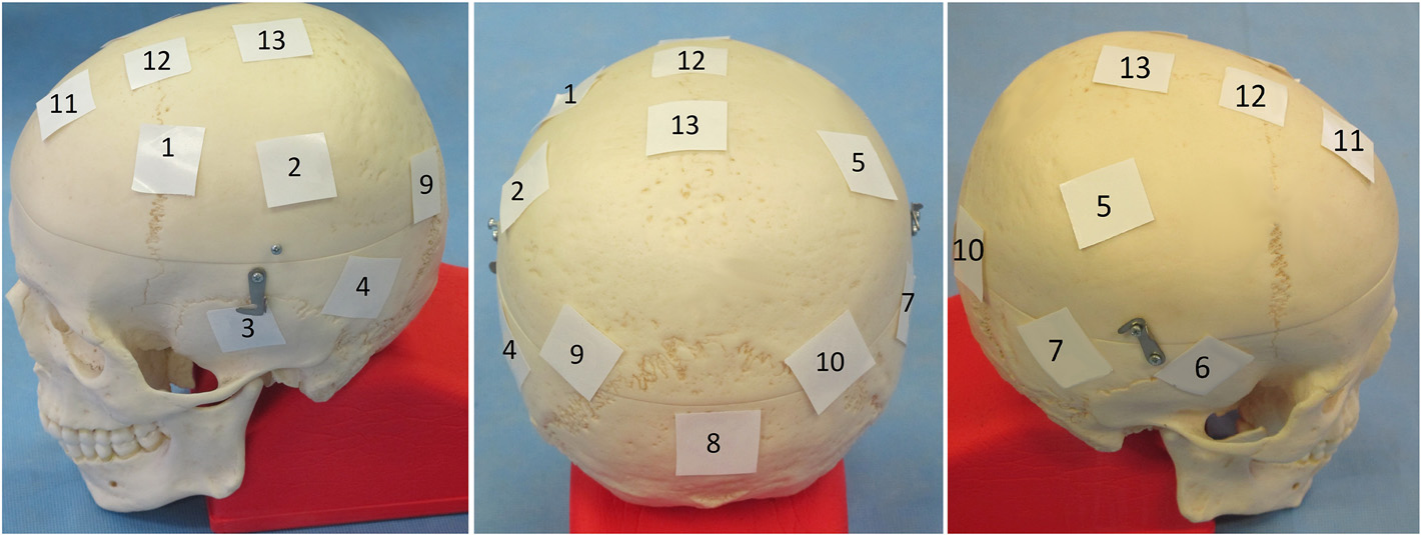
Distribution of the film measurement locations across the scalp.

Thirteen radiochromic films (2.2 × 2.2 cm^2^, EBT2 lot nr. A06271203, Gafchromic, Ashland Specialty Ingredients, Wayne, NJ, USA) were implanted equally distributed across the cadaver scalp (Figure 2). They were protected from the cadaver’s embalmment fluid by wrapping them in a plastic paraffin film casing (Parafilm, Pechiney Plastic Packaging Company, Chicago, Illinois). As the internal structure of the cadaver skin cannot be discerned macroscopically, all films were implanted subcutaneously. Consequently, their implantation depth always exceeded the hair follicle depth. Moreover, all films were positioned within the build-up region with doses generally rising with depth. Therefore, we assume their measured doses to serve as an estimation and upper limit for the dose to the hair follicles. The implantation depth was estimated using CT images and ranged from 2.0 to 11.7 mm.

Radiochromic films were digitized on an Epson Expression 10000XL flatbed scanner (Seiko Epson Corporation, Suwa, Nagano, Japan) and analyzed using the net optical density (OD) in the red color channel [21]. The mean doses and dose-volume-histograms were computed for the complete films, only excluding a narrow strip along the film’s edge and the small orientation markers on the film. All reported relative doses and dose differences are normalized to both plans’ prescription dose (per fraction) of 4 Gy.

The films were not present in the cadaver during planning CT imaging. Instead, the visibility of their positions was enhanced by replacing them with 2.2 × 2.2 × 0.02 cm^3^ aluminum plates. We observed no impaired image distortion for the applied CT settings. The films were exposed to the kV-irradiation of the CBCT cadaver during positioning procedures. The impact of these procedures on the films’ OD was assessed by exposing one set of films to the CBCT irradiation only. As the CBCT induced film response corresponded to only 0.56% of the prescription dose on average, the CBCT-related film darkening was neglected in further experiments.

#### *Dose calculation accuracy*

The dose calculation accuracy of the collapsed-cone convolution (CCC) algorithm implemented in Pinnacle was evaluated by comparing the measured and calculated data. To that purpose, the outline of the aluminum plates in the CT images served for contouring the location of the radiochromic films. The density within these contours was overridden with the density of the surrounding dermal tissue (1.07 g/cm^3^). As the calculation grid is generally considered a determinative parameter for dose calculation accuracy in the build-up region [14], the calculations were performed for both the clinically used 4 mm, as well as for a 1 mm calculation grid. Within these computations, the fluence density grid always equaled the calculation grid. The mean absorbed doses and dose-volume-histograms were then computed for all radiochromic film positions.

### Phase II study

#### *Patients and procedures*

Eligible patients who presented at the department of radiation oncology of Ghent University Hospital were recruited for the study. Eligibility requirements were: age *≥18,* brain metastases eligible for WBRT: recursive partitioning analysis (RPA) class I or II and > 3 brain metastases or RPA class III or meningitis carcinomatosa, and signed written informed consent. Patients were excluded if they received prior cranial radiotherapy, had pre-existing alopecia or were diagnosed with leukemia, lymphoma or germ-cell tumor. The study was approved by the local Institutional Review Board (reference number: EC2011/504) and registered at clinicaltrials.gov: NCT01421316.

All patients received VMAT-WBRT for brain metastases detected with brain contrast-enhanced computed tomography and/or magnetic resonance imaging within 1 month before enrolment. The delineation, treatment planning and delivery for the VMAT technique has been described above. The primary endpoint was the mean score of hair loss at 1 month after VMAT-WBRT scored with the European Organization for Research and Treatment of Cancer (EORTC) Quality of Life Questionnaire Brain Cancer Module (EORTC-QLQ-BN20) [22]. The EORTC-QLQ-BN20 is designed for use with patients undergoing chemotherapy or radiotherapy, and is composed of 20 questions assessing visual disorders, motor dysfunction, communication deficit, various disease symptoms (e.g., headaches and seizures), treatment toxicities (e.g., hair loss), and future uncertainty. This questionnaire was used as a supplement to the EORTC-QLQ-C15-PAL [23]. The questions on both measures were scaled, scored and reported using the recommended EORTC procedures [24,25]. As a secondary endpoint, the global alopecia areata severity score was used (combination of extent and density of scalp hair loss) [16]. The total score is calculated by visually determining the amount of terminal hair loss in each of the four views of the scalp and adding these together with a maximum score of 100% corresponding with complete alopecia. This global severity score may be called the “Severity of Alopecia Tool” or SALT score. The four views of the scalp were photographed in standardized conditions before radiotherapy and 1 month after radiotherapy [16].

#### *Sample size and statistical analysis*

At 1 month following OF-WBRT, the mean score of hair loss (EORTC BN20) is 36.7 (out of 100, with a standard deviation of 17) compared to 11.7 in an untreated patient group [6]. We hypothesize that with VMAT-WBRT, the mean score of hair loss will be 15 points lower compared to OF-WBRT [25]. Differences of at least 10 points (on a 0 to 100 scale) are classified as the minimum clinically meaningful change in the mean value of a HRQOL parameter. Mean changes of more than 20 points were classed as large effects. To detect a significant result at the 5% level, with 80% power we would need to treat 21 patients in this single arm study. With an estimated dropout of 20%, due to the median prognosis of 2 months, we aimed to include 26 patients. Descriptive statistics were used to calculate means and standard deviations. The QOL and SALT score results are presented as mean scores and were compared between time points using the Wilcoxon signed rank test (p < 0.05 considered significant). An interim analysis was planned after including 10 patients to rule out futility, defined as a mean score of hair loss exceeding 56.7.

## Results

### Dosimetric evaluation

**Figure 3:**
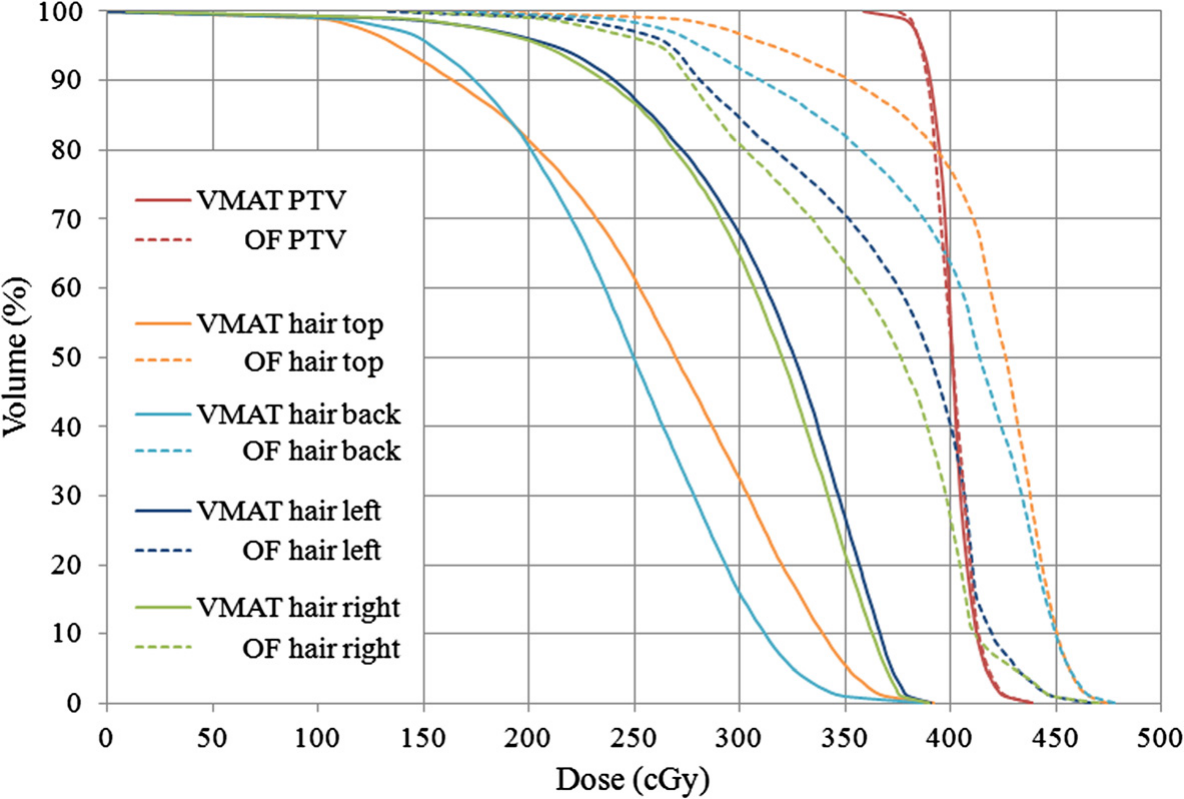
DVH’s for the VMAT- and OF- WBRT cadaver plans calculated with a 1 mm dose grid for 1 fraction (prescription dose 4 Gy).

The cadaver’s planning results are depicted in Figure 3, demonstrating the DVH’s calculated with a 1 mm dose grid, and Table 1, showing the corresponding calculated median doses. Compared to the OF-plan, the VMAT computations clearly predict a mean dose decrease of approximately 25% in the total hair follicle volume. The highest reductions are found in the hair top and back subvolumes, where a high-dose region in the OF-plan is transformed into a low dose region for the VMAT-plan.

**Table 1:** Calculated median doses (D50) for the hair follicle volume and its subvolumes for the VMAT- and OF- plan normalized to the prescription dose per fraction (4 Gy)

	**VMAT-WBRT**		**OF-WBRT**	
**Hair follicle subvolume**	**Calculated D50**	**Calculated D50**	**Calculated D50**	**Calculated D50**
	**1 mm grid (%)**	**4 mm grid (%)**	**1 mm grid (%)**	**4 mm grid (%)**
Total	72.4	68.5	100.10	92.34
Top	67.3	60.8	106.60	90.79
Left	81.4	78.4	97.58	92.92
Back	62.3	59.8	103.40	94.09
Right	80	75.9	93.99	89.82

As illustrated in Table 2, our radiochromic film measurements conclusively confirmed these predicted tendencies. For all films in the VMAT-plan, an important dose reduction was found compared to the OF-plan, with an average film dose reduction of 20.5%. As predicted by the TPS, the OF-plan demonstrated a remarkable high-dose region on the hair top and back subvolumes (mean dose 112.2% for films 8, 11–13), which is transformed into a low dose region in the VMAT-plan (mean dose 70.4%).

**Table 2:** mean measured and calculated doses for all films implanted subcutaneously in the cadaver, normalized to the prescription dose per fraction (4 Gy)

		**VMAT-WBRT**				**OF-WBRT**		
**Hair follicle subvolume**	**Film nr.**	**Depth (mm)**	**Measured dose (%)**	**Calculated dose 1 mm grid (%)**	**Calculated dose 4 mm grid (%)**	**Measureddose (%)**	**Calculated dose1 mm grid (%)**	**Calculated dose 4 mm grid (%)**
Left	1	2.53	91.0	89.0	85.3	103.8	94.1	91.1
	2	5.74	85.6	85.0	82.1	101.1	98.5	94.2
	3	9.74	85.9	89.6	88.3	94.1	98.3	95.0
	4	6.34	77.9	78.0	77.2	98.0	105.9	97.0
	*Mean*	6.09	85.1	85.4	83.2	99.2	99.2	94.3
Right	5	11.65	94.5	85.0	82.9	97.9	103.9	96.7
	6	6.73	96.5	91.0	89.0	94.9	99.8	95.8
	7	8.8	90.2	81.7	81.1	98.5	102.9	97.0
	*Mean*	9.06	93.7	85.9	84.4	97.1	102.2	96.5
Back	8	7.24	71.2	69.7	69.1	110.4	109.9	103.3
	9	2.53	83.9	79.8	78.6	100.0	101.7	96.8
	10	11.04	81.0	85.7	85.1	98.5	102.7	97.8
	*Mean*	6.94	78.7	78.4	77.6	102.9	104.8	99.3
Top	11	4.68	75.2	70.4	64.2	114.2	111.8	91.5
	12	2.02	84.3	81.1	70.4	112.3	111.4	89.4
	13	2.03	51.1	59.7	56.9	111.9	108.9	99.7
	*Mean*	2.91	70.2	70.4	63.9	112.8	110.7	93.5
All films	Mean	6.24	82.2	80.4	77.7	102.7	103.8	95.8

The corresponding standard deviations, representing dose variations within each film, amounted to approximately 3.5% and 1.9% for the VMAT- and OF- films respectively. As the dose-volume-histograms for all detectors equally showed little dose variation within each film, further data analysis was uniquely based on the mean dose per film.

The calculation accuracy of the mean dose of the film was found to be similar for both techniques, except for the films on the frontal-vertex-occipital medial axis. For the clinically used 4 mm calculation grid, the mean dose discrepancy between measurements and calculations equaled 6.9% and 6.4% for the OF- and VMAT-plan respectively. In the OF-plan, however, the use of this 4 mm grid resulted in remarkable accuracy degradation on the frontal-vertex-occipital medial axis. The high dose in this region was then underestimated by 16.22% on average and 22.9% at maximum. In the corresponding lower dose region in the VMAT-plan, this tendency was much less pronounced, demonstrating a mean dose discrepancy of 8.2% only. The use of a 1 mm calculation grid size resolved this issue. The average dose discrepancy then amounted to 4.3% and 4.1% for the VMAT- and OF- plan respectively with a maximum discrepancy of 3.2% on the frontal-vertex-occipital medial axis.

### Phase II trial

A total of 10 patients were included between 26/10/11 and 25/05/2012 before the trial was halted due to futility at time of the interim analysis. We treated 6 male and 4 female patients; with a median age of 63 years (range 45–72). The primary tumour site was lung (n = 5), pancreas (n = 2), breast, prostate and colon. All patients were RPA class I (n = 5) or II (n = 5). The median number of metastases treated was 4 (range: 1–7).

**Figure 4:**
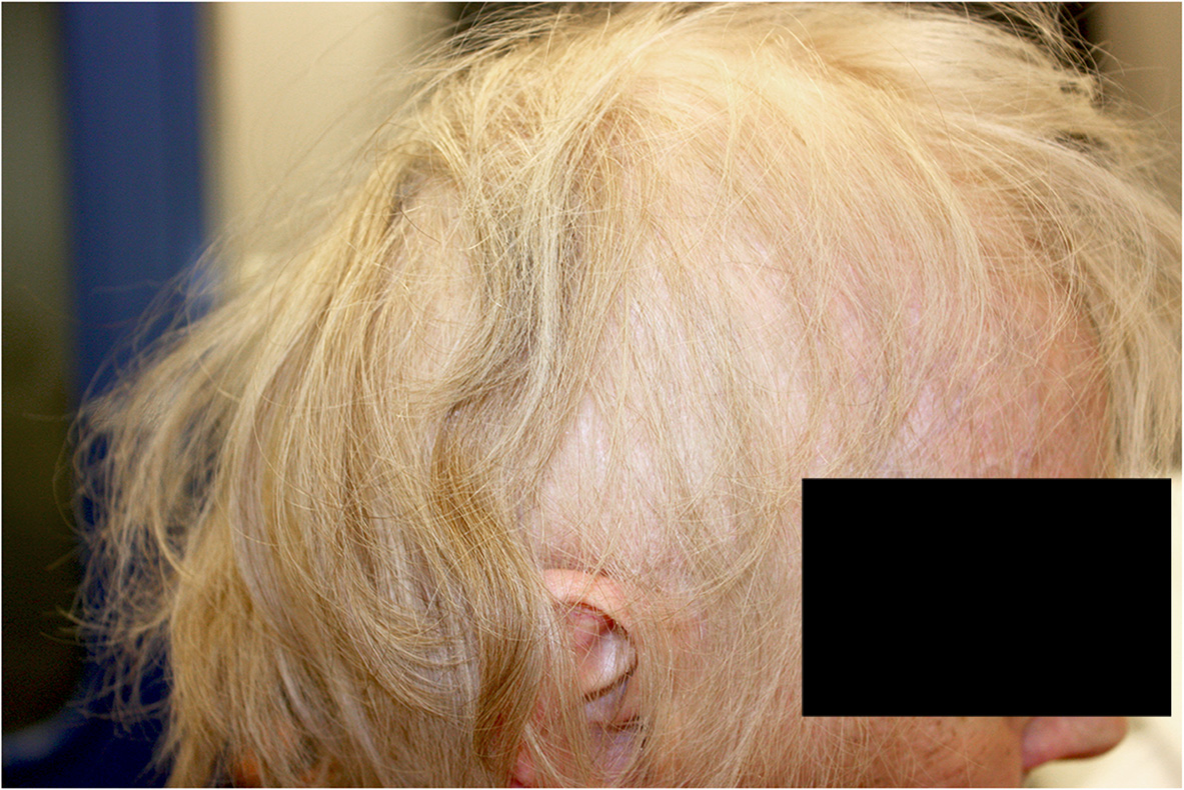
Lateral view of a patient’s scalp with a 55% hair loss according to the SALT-score, but scoring 100 on the EORTC hair loss scale.

As illustrated in Table 3, the average median dose to the hair follicle volume was 12.6 Gy (SD 0.9), which corresponds to a 37% dose reduction compared to the prescribed dose of 20 Gy. For the 4 subvolumes of the hair follicle volume, the dose was reduced with 22%, 20%, 44% and 48% for the left side, right side, top of head and back of head, respectively. For all hair follicle subvolumes, the mean doses predicted by the TPS for the patient group differed by only 7.3% at maximum from the corresponding doses calculated for the cadaver.Two patients died before reaching 1 month follow-up. For the remaining 8 patients, the mean score of hair loss, as measured with the EORTC BN20 questionnaire prior to WBRT, was 9.5 (SD 16.2) compared to 95 (SD 12.6) after WBRT (p = 0.01). The mean total SALT score before WBRT was 0 (SD 0) compared to 75 (SD 13) after WBRT, corresponding to 75% hair loss. One patient was excluded from the photographic scoring after shaving his head. When sub-stratifying the score according to the four subvolumes, we observed a mean hair loss of 96%, 96%, 65% and 60% at the left side, right side, top of scalp and back of scalp, respectively (p = 0.04). A lateral view of a patient with 55% hair loss according to the SALT- score is depicted in Figure 4.

**Table 3:** Patient planning results of the phase II trial for the different hair follicle subvolumes for 5 fractions (prescription dose 20 Gy)

**Hair follicle subvolume**	**Mean SALT score**	**Mean D50 ± SD (Gy)**	**% of prescribed dose**	**Mean V5 ± SD (%)**	**Mean V10 ± SD (%)**	**Mean V15 ± SD (%)**	**Mean V20 ± SD (%)**
Total	75 ± 13	12.6 ± 0.9	62.9	92.2 ± 4.2	66.8 ± 6.4	31.1 ± 9.3	0.4 ± 0.8
Left	96 ± 0.05	15.7 ± 0.8	78.4	86.1 ± 1.5	79.8 ± 8.5	51.6 ± 8.5	0 ± 0
Right	96 ± 0.05	16.0 ± 0.9	80.0	85.6 ± 2.3	78.9 ± 4.5	50.5 ± 7.1	0 ± 0
Back	60 ± 18.7	10.5 ± 1.3	52.3	77.9 ± 6.8	45.0 ± 10.6	12.8 ± 9.2	0 ± 0
Top	65 ± 18.3	11.3 ± 2.7	56.3	76.6 ± 11.3	49.9 ± 18.4	21.8 ± 16.8	0.6 ± 2

## Discussion

In literature, the interpretation of data on intensity modulated techniques in hair-sparing WBRT [10,11] has been impeded by the lack of knowledge on the dose-effect relation of temporary alopecia. Additionally, plan optimization and evaluation in the hair follicle region is limited by known inaccuracies of the TPS in calculating build-up doses [14]. As a consequence, the available dosimetric studies by *Roberge* et al. [11] and *Mancini* et al. [10] do not allow to predict these techniques’ impact on temporary alopecia. This investigation is, to our knowledge, the first study to overcome these issues by quantitatively relating dose measurements and planning data to clinical results on temporary alopecia in WBRT.

In this respect, our dose measurements demonstrated the potential of VMAT-WBRT to reduce the subcutaneously absorbed dose by 20.5%. *Roberge* et al., for comparison, measured a dose reduction of 53% at 1 mm depth with TLD’s and calculated a dose reduction of 38% within the first 5 mm of the skin when comparing IMRT to OF-WBRT [11]. Note, however, that our study reports the subcutaneously absorbed dose, serving as an upper limit for hair follicle dose, rather than the dose measured at the surface by *Roberge* et al.. Consequently, as the sparing of any organ at risk depends on its distance to the target volume, the sparing of the more superficially positioned hair follicles will most likely exceed rather than be smaller than our reported values [11]. Interestingly, the lowest doses in VMAT-WBRT were found on the top of the scalp, where high doses typically occur in OF-WBRT. These high OF-doses might be attributed to the extremely oblique and overlapping incidence of opposed beams, thus boosting surface dose and degrading the modeling accuracy of the TPS. The use of arc techniques obviously mitigates this effect by spreading it out across the scalp. Combined with the concomitant lack of exit dose in this region compared to the remainder of the scalp, this effect results in a low dose region in the VMAT-plan. A more optimized OF-WBRT could probably improve the doses to the hair follicle region, when using portals shaped around the PTV, the use of compensation and higher energy photons.

Most importantly, our phase II trial provided valuable insight into the impact of these dose reductions on the risk of temporary alopecia. As for the human cadaver, the patients’ doses in the hair follicle left and hair right subvolumes were predicted to be around 15–17 Gy for 5 fractions respectively and were related to 96% hair loss. However, as for the cadaver, the TPS equally predicted a low dose region of 10.5-11.3 Gy on the frontal-vertex-occipital medial axis (top and back subvolumes), resulting in 60 to 65% hair loss. Based on these data, we hypothesize that the threshold TPS dose for temporary alopecia is around or slightly lower than 10 Gy in 5 fractions, as some but not all of the patients retained their hair in this region. In contrast, in the abstract of *Ting* et al. a mean calculated scalp dose of 18.5 Gy (within the first 3 mm of tissue underlying the skin) delivered in 10–15 fractions in IMRT-WBRT was related to subjectively reduced hair loss in all patients. Half of the patients demonstrated no noticeable hair loss, while the other half experienced subjectively mild hair loss. However, they neither quantitatively scored hair-loss nor assessed QOL, making it difficult to compare results.

Although some patients retained their hair on the top and back of the head, this was not related to an improved QOL. Following a pre-planned interim analysis, the rules for futility were met, with a mean score of hair loss exceeding 56.7 on the EORTC BN20 (mean score of 95). The score of hair loss in our study was higher than in the literature, where in general scores between 30 and 40 are reported [4,6,26,27]. In the study of *Steinman* et al. and *Slotman* et al., 42% and 22% of patients showed an increase in hair loss scores exceeding 20 points, respectively, compared to all patients in our study. In a recent study of VMAT-WBRT with simultaneous integrated boost for brain metastases, the mean score of hair loss was 23.8 [28], which is lower compared to literature of OF-WBRT and in range of our primary objective to lower the score to 21.7. We hypothesize that the worse score in the current study compared to literature is due to the patients’ knowledge of the primary goal of VMAT-WBRT to reduce hair loss. This discordance is reflected in Figure 4, showing a lateral view of a patient scoring 100 on the EORTC hair loss scale although hair loss was only 55% according to the SALT score.

Further improvements to reduce alopecia might require the combination of a reduced dose to the hair follicles in combination with radioprotectors designed to reduce the damage to the normal tissues caused by radiation, such as nitroxides, antioxidants [29]. As an alternative, the dose to the uninvolved brain might be decreased to reduce toxicity. This approach is currently tested with hippocampal sparing to avoid memory loss [30]. However, a dose reduction to the peripheral zone of the brain to avoid alopecia should be approached carefully as most brain metastases are located in the outer cortex of the brain [30].

## Conclusion

Compared to OF-WBRT, VMAT-WBRT substantially reduces hair follicle dose, especially on the frontal-vertex-occipital medial axis. Our phase II trial was halted due to futility with a EORTC BN20 hair loss score of 95 (SD 12.6) and a mean SALT score of 75.

## Competing interests

The authors declare that they have no competing interests.

## Authors’ contributions

ADP and PO had full access to all the data in the study and takes responsibility for the integrity of the data and the accuracy of the data analysis. Study concept and design: ADP and PO. Acquisition of data: ADP, JVDV, BS, AG, TVH, TB. Analysis and interpretation of data: ADP and PO. Drafting of the manuscript: ADP, PO, WDN. Critical revision of the manuscript for important intellectual content: TV and CDW. Statistical analysis: ADP and PO. Supervision: PO. All authors read and approved the final manuscript.
